# On Sudden Propensity to Murder and Suicide

**Published:** 1831-01-01

**Authors:** 


### A CASE OF SUDDEN PROPENSITY TO MURDER AND SUICIDE. By Dr. OTTO of Copenhagen.

FREDERICK JENSEN, workman, 37 years old, had for some time suffered from fits of giddiness, which always obliged him to keep hold of the nearest objects. In the spring of 1828 he lost a beloved daughter, which afflicted him very much. The state of his health was nevertheless *perfect, in mind as well as in body*, when he one day (Sunday the 28th of September, 1828), after dinner, told his wife that he would take a walk with his son, a boy ten years old. He did so, and went with him to the green which encircles the citadel. When he came there,—he now relates,—"a strange confusion came over him;" it appeared like a matter of absolute necessity to him to drown his son and himself in the waters at the citadel. Quite unconscious of what he was doing, he ran towards the water with the boy in his hand. A man, surprised at his behaviour, stopt him there, took the boy from him, and tried to persuade him to leave the water; but he became angry, and answered, that he intended to take a walk, and asked, "Whether any body had a right to forbid him to do so?" The man left him, but took the boy along with him. An hour after, he was drawn out from the water, into which he had thrown himself, and taken to prison. As he still shewed symptoms of insanity, he was bled and purged, and, two days after, was brought into the hospital, and committed to the care of my friend Dr. Wendt, who has perfectly cured him, and who kindly afforded me the opportunity to see and to speak with the patient.

He now very quietly tells the whole event himself, but is not able to explain the cause of the suddenly rising desire to kill himself and the boy, whom he loved heartily. This cause is only to be sought in congestion of blood to the brain, the same which before had caused his giddiness; and, whether we adopt an organ of Destructiveness in the brain or not, it is to be assumed, that the propensity to kill himself and the son arose from a morbid excitation of a certain part of the brain. The disposition to congestion originated from a fall he suffered on the head in the year 1820.

We ask, whether any body in this case would have admitted responsibility of crime, if the patient really had executed his plan to murder the son?

In concluding, we beg permission to mention some very remarkable pathological phenomena in this man. Since he fell on his head in 1820 (on the right side of it), all the *right* side of his body is become considerably leaner than the *left;* so that the *right* arm is *not half* so thick as the *left;* the *left* ribs are much less prominent than the *right* ones; and—what is still more remarkable—the whole *right* half part of the head is evidently less than the *left* one. —*Phrenological Journal.*

---

